# IL-22/STAT3-Induced Increases in SLURP1 Expression within Psoriatic Lesions Exerts Antimicrobial Effects against *Staphylococcus aureus*

**DOI:** 10.1371/journal.pone.0140750

**Published:** 2015-10-16

**Authors:** Yasuhiro Moriwaki, Kiyoko Takada, Toshinori Nagasaki, Natsuki Kubo, Tomohiro Ishii, Kazuaki Kose, Taihei Kageyama, Shoutaro Tsuji, Koichiro Kawashima, Hidemi Misawa

**Affiliations:** 1 Department of Pharmacology, Faculty of Pharmacy, Keio University, Minato-ku, Tokyo 105–8512, Japan; 2 Molecular Diagnostics Project, Kanagawa Cancer Center Research Institute, Yokohama, Kanagawa, Japan; 3 Department of Molecular Pharmacology, Kitasato University School of Pharmacy, Minato-ku, Tokyo, Japan; INSERM-Université Paris-Sud, FRANCE

## Abstract

**Background:**

*SLURP1* is the causal gene for Mal de Meleda (MDM), an autosomal recessive skin disorder characterized by diffuse palmoplantar keratoderma and transgressive keratosis. Moreover, although SLURP1 likely serves as an important proliferation/differentiation factor in keratinocytes, the possible relation between SLURP1 and other skin diseases, such as psoriasis and atopic dermatitis, has not been studied, and the pathophysiological control of *SLURP1* expression in keratinocytes is largely unknown.

**Objectives:**

Our aim was to examine the involvement of SLURP1 in the pathophysiology of psoriasis using an imiquimod (IMQ)-induced psoriasis model mice and normal human epidermal keratinocytes (NHEKs).

**Results:**

SLURP1 expression was up-regulated in the skin of IMQ-induced psoriasis model mice. In NHEKs stimulated with the inflammatory cytokines IL-17, IL-22 and TNF-α, which are reportedly expressed in psoriatic lesions, *SLURP1* mRNA expression was significantly up-regulated by IL-22 but not the other two cytokines. The stimulatory effect of IL-22 was completely suppressed in NHEKs treated with a STAT3 inhibitor or transfected with siRNA targeting STAT3. Because IL-22 induces production of antimicrobial proteins in epithelial cells, the antibacterial activity of SLURP1 was assessed against *Staphylococcus aureus* (*S*. *aureus*), which is known to be associated with disease severity in psoriasis. SLURP1 significantly suppressed the growth of *S*. *aureus*.

**Conclusions:**

These results indicate SLURP1 participates in pathophysiology of psoriasis by regulating keratinocyte proliferation and differentiation, and by suppressing the growth of *S*. *aureus*.

## Introduction

Psoriasis is a commonly occurring inflammatory skin disorder, affecting approximately 3% of the population worldwide. Psoriasis is characterized by over-proliferation and/or disturbed differentiation of keratinocytes [1], as well as by enhanced expression of tumor necrosis factor-α (TNF-α), IL-17 and IL-22 within the psoriatic skin [2–4]. Activated CD4^+^ and CD8^+^ T cells populate the psoriatic plaques [5], and symptoms of the disease are ameliorated by drugs targeting T cells [6]. These findings suggest that the abnormal turnover of keratinocytes in psoriasis is caused by cytokines secreted by activated T cells within psoriatic plaques.

Mal de Meleda (MDM; OMIM2483000) is a rare autosomal recessive skin disorder characterized by diffuse palmoplantar keratoderma (PPK) and transgressive keratosis with an onset in early infancy. As with psoriasis, it is suspected that MDM is related to defective T-cell function [7]. Homozygous mutations of the *SLURP1* gene (previously known as *ARS component B*), encoding secreted lymphocyte antigen-6/urokinase-type plasminogen activator receptor related protein-1 (SLURP1), have been identified as a cause of MDM [8]. SLURP1 is a secreted protein that assumes the three-finger folded structure found in a family of neurotoxins from snakes or frogs [9], and reportedly accelerates differentiation and apoptosis in keratinocytes [10]. Furthermore, *SLURP1*-deficient mice show severe palmoplantar keratoderma characterized by increased keratinocyte proliferation and water barrier defects [11]. SLURP1 also acts as an allosteric agonist that enhances acetylcholine (ACh)-evoked macroscopic currents in Xenopus oocytes expressing recombinant human α7-nicotinic acetylcholine receptors (α7-nAChRs) [12]. α7-nAChRs reportedly control skin homeostasis and the terminal differentiation of epidermal keratinocytes required for formation of the skin barrier [13]. These effects of SLURP1 on the skin suggest SLURP1 may be involved in regulating the proliferation and differentiation of keratinocytes, not only in MDM but also in other skin disorders, including psoriasis.

*Staphylococcus aureus* (*S*. *aureus*) is detected in about 50% of skin lesions in patients with psoriasis [14,15] and is associated with a higher Psoriasis Area Severity Index (PASI) score [16]. In addition, Niebuhr et al. [17] reported that staphylococcal enterotoxin B (SEB) and α-toxin from *S*. *aureus* induce secretion of IL-22 from peripheral blood mononuclear cells and CD4^+^ T cells, and several reports have shown that IL-22 production is associated with psoriasis [3,18,19]. IL-22 is an IL-10 family cytokine that acts mainly on epithelial cells [20]. Within the skin, IL-22 mediates keratinocyte proliferation and epidermal hyperplasia, inhibits terminal differentiation of keratinocytes, and induces production of antimicrobial proteins (AMPs) [19,21,22]. IL-22-induced AMPs such as β-defensin2, S100A7 and CAP18/LL37 all have the ability to suppress growth of *S*. *aureus* [23,24].

In the present study, we explored the involvement of SLURP1 in the pathophysiology of psoriasis using imiquimod (IMQ)-induced psoriatic model mice and normal human epidermal keratinocytes (NHEKs). We found that SLURP1 expression is markedly increased via STAT3 signaling in psoriatic skin, and that recombinant SLURP1 suppresses the growth of *S*. *aureus*. These results suggest SLURP1 expression is controlled by IL-22 and is involved in the maintenance of skin homeostasis as well as the pathogenesis of psoriasis.

## Materials and Methods

### Mice and treatments

All experiments were reviewed and approved by the Keio University Animal Care and Use Committee, and care was taken to minimize suffering and limit the number of animals used.

Female BALB/c mice were purchased from Sankyo Labo Service (Tokyo, Japan). IMQ-induced skin inflammation was induced as described previously [25]. In brief, 8- to 11-week-old mice received a daily topical dose of 62.5 mg of commercially available IMQ cream (5%: a daily dose of 3.125 mg of the active compound, Beselna Cream; Mochida Pharmaceutical Co., Ltd., Shizuoka, Japan) on their shaved back for 2 or 4 consecutive days. Control mice were treated similarly with a control vehicle cream.

### Cell culture, treatment with pharmacological inhibitors and transient transfection of siRNAs

Second-passage neonatal foreskin NHEKs were purchased from Kurabo Industries (Osaka, Japan) and cultured in serum-free HuMedai-KG2 keratinocyte growth medium (Kurabo Industries) containing human epidermal growth factor (0.1 ng/ml), insulin (10 μg/ml), gentamicin (50 μg/ml), amphotericin B (50 ng/ml) and bovine pituitary extract (0.4%, v/v) at 37°C under an atmosphere of 95% air/5% CO_2_. Cells were passaged at 60–70% confluence to avoid differentiation, and the experiments were conducted using subconfluent passage 3 cells in the proliferative phase at 60–80% confluence.

The following pharmacological inhibitors and concentrations were used: the STAT3 inhibitor S3I-201 (50 μM, BioVision, Bilpitas, CA, USA), the STAT5 inhibitor 573108 (100 μM, Merck Millipore, Billerica, MA, USA), the MAP kinase kinase (also known as MAPK/ERK kinase or MEK kinase) inhibitor PD98059 (30 μM, Cayman, Chemical Co, Ann Arbor, MI, USA), cycloheximide (0.3 μg/mL, Sigma-Aldrich, St. Louis, MO, USA) and actinomycin D (0.25 μg/mL, Sigma-Aldrich).

NHEKs were seeded into 6-well plates at a density of 2 × 10^4^ cells/well and cultured for 5 days. Using Lipofectamine 2000 (Life Technologies, Carlsbad, CA, USA) according to the manufacturer’s instructions, the cells were then transfected with 100 nM siRNA targeting *STAT3* (Cell Signaling Technology, Inc., Danvers, MA, USA) or *STAT5* (Cell Signaling Technology, Inc.), or with a scrambled control siRNA. Forty-eight hours after siRNA transfection, NHEKs were transferred to growth medium with or without 50 ng/mL IL-22, and were cultured for another 24 h.

### Real-time Quantitative PCR

Total mRNA was extracted from back skin harvested after sacrificing the mice or from NHEKs using an acid guanidinium thiocyanate phenol-chloroform extraction method with ISOGENE II (Nippon Gene Co., Ltd., Tokyo, Japan). First-strand cDNA was synthesized from 1 μg of total RNA using PrimeScript RT Master Mix (TaKaRa Bio Inc., Shiga, Japan). Quantitative PCR was then performed in triplicate in a total reaction volume of 20 μL using Thunderbird SYBR qPCR Mix (Toyobo Co., Ltd., Osaka, Japan) and 2 μL of cDNA per reaction in a 96CFX Real-Time PCR Detection System (Bio-Rad Laboratories, Inc., Hercules, CA, USA). PCR was performed using a 2-step protocol. Initial denaturation of the cDNA at 95°C for 3 min was followed by 40 cycles of 95°C for 3 s and 60°C for 30 s. The primers used for amplification were as follows: for mouse *IL-22*, 5'-TTGAGGTGTCCAACTTCCAGC-3' (sense) and 5'-AGCCGGACATCTGTGTTGTTA-3' (antisense); for mouse *SLURP1*, 5'-TTCCGTGACCTCTGCAACTC-3' (sense) and 5'-AGCTTGGTGGACAGTGAGTG-3' (antisense); for mouse *GAPDH*, 5'-TGTGTCCGTCGTGGATCTGA-3' (sense) and 5'-TTGCTGTTGAAGTCGCAGGAG-3' (antisense); for human *SLURP1*, 5'-GTGAGGCCCTCAAGTGCTAC-3' (sense) and 5'-GCTCTGGTTGAAGGGGTACTC-3' (antisense); for human *CCL5*, 5'-TACACCAGTGGCAAGTGCTC-3' (sense) and 5'-TGTACTCCCGAACCCATTTC-3' (antisense); for human *S100A7*, 5'-GCTGACGATGATGAAGGAGAACT-3' (sense) and 5'-GTAATTTGTGCCCTTTTTGTCACA-3' (antisense); and for human *GAPDH*, 5'-AATCCCATCACCATCTTCCA-3' (sense) and 5'-TGGACTCCACGACGTACTCA-3' (antisense). Relative mRNA levels were calculated using the *-ΔΔCt* method with *GAPDH* as an internal control. At least six independent experiments were performed.

### Western blot and Antibodies

Western blot analysis was performed using standard techniques as described previously [26]. Briefly, the cells were lysed in lysis buffer (20 mM HEPES, pH 7.4, 150 mM NaCl, 10% glycerol, 1% Triton X-100) supplemented with Complete Protease Inhibitors (Roche Diagnostics, Mannheim, Germany). Aliquots of lysate containing 30 μg of protein were subjected to SDS-polyacrylamide gel electrophoresis, after which the proteins were transferred to polyvinylidene difluoride (PVDF) membranes (Immobilon-P; Merck Millipore). The membranes were then probed first with specific primary antibodies and then with the appropriate HRP-conjugated secondary antibodies (Bio-Rad Laboratories, Inc.). Immunopositive proteins were detected using ECL Western Blotting Detection Reagent (GE Healthcare, Madison, WI, USA). As a loading control, the membrane was probed with a monoclonal antibody against β-actin (C4, 0.5 μg/mL; Chemicon, Temecula, CA, USA). The antibodies used in this study were monoclonal anti-human SLURP1 (1:1000 dilution; Abnova, Taipei, Taiwan), polyclonal anti-human STAT-3 (1:1000 dilution; Santa Cruz Biotechnology, Dallas, TX, USA), and monoclonal anti-human STAT-5 (1:1000 dilution; Santa Cruz Biotechnology).

### Immunohistochemistry

BALB/c mice were deeply anesthetized with sodium pentobarbital and then perfused through the aortic cone with phosphate-buffered saline (PBS) followed by 4% paraformaldehyde (PFA) in 0.1 M phosphate buffer (PB) at pH 7.4. Thereafter, skin from the shaved back of each mouse was removed and post-fixed in the same fixative overnight at 4°C, after which they were immersed in 20% sucrose in PB overnight at 4°C. The tissues were then frozen in OCT, cut into 15-μm sections on a cryostat, and thaw-mounted on MAS-coated slides (Matsunami Glass, Osaka, Japan). Immunofluorescent labeling was performed using our rabbit polyclonal anti-SLURP1 antibody (0.7 μg/ml; 26). Alexa Fluor 488-conjugated goat anti-rabbit IgG (1:200 dilution; Life Technologies) was used as the secondary antibody. Sections were then wet-mounted in Fluoremount-G (SouthernBiotech, Birmingham, AL, USA) and examined using an Olympus FV-1000 confocal microscope system (Tokyo, Japan) equipped with a 60x objective lens (N.A. = 1.35). Confocal images of skin sections were acquired using a single sectioning.

### *In vitro* antibacterial activity assay

RK13 cells were cultured in Dulbecco’s modified Eagle’s medium supplemented with 10% heat-inactivated fetal bovine serum (ICN Biomedical, Inc., Cleveland, OH, USA), 50 U/ml penicillin and 50 U/ml streptomycin at 37°C under an atmosphere of 95% air/5% CO2. Plasmids encoding human SLURP1 cDNAs were transfected into cells using Lipofectamine 2000 according to the manufacturer’s instructions. After incubating the transfectant cells for 72 h, the culture supernatants were collected and recombinant human (rh)SLURP1 was partially purified using an anti-human SLURP1 antibody-conjugated column. α-defensin2 and magainin1 were purchased from Peptide Institute, Inc. (Osaka, Japan) and used as a positive control.

The bacterial strains used for antibacterial activity assays were *S*. *aureus* 209P and *Escherichia coli* (*E*. *coli*). Bacterial cultures were grown to mid-exponential growth phase, and the cell concentrations were photometrically estimated based on the OD_620_. After dilution to a concentration of 1 x 10^4^ CFU/ml, 50-μl aliquots of the dilutions were incubated at 37°C for 18 h with 50 μl of the respective protein samples in a total volume of 100 μl in a sterile 96-well microtiter plate (Corning Inc., Corning, NY, USA). Inhibition of bacterial growth was evaluated by comparing the change in the OD_620_ using a microplate reader (TECAN-200, Tecan Trading AG, Männedorf, Switzerland). The results are presented as the percentage of growth relative to the untreated control. This assay was repeated in six independent experiments performed in triplicate.

## Results

### Increased SLURP1 expression in the skin of psoriatic model mice

**Fig 1:**
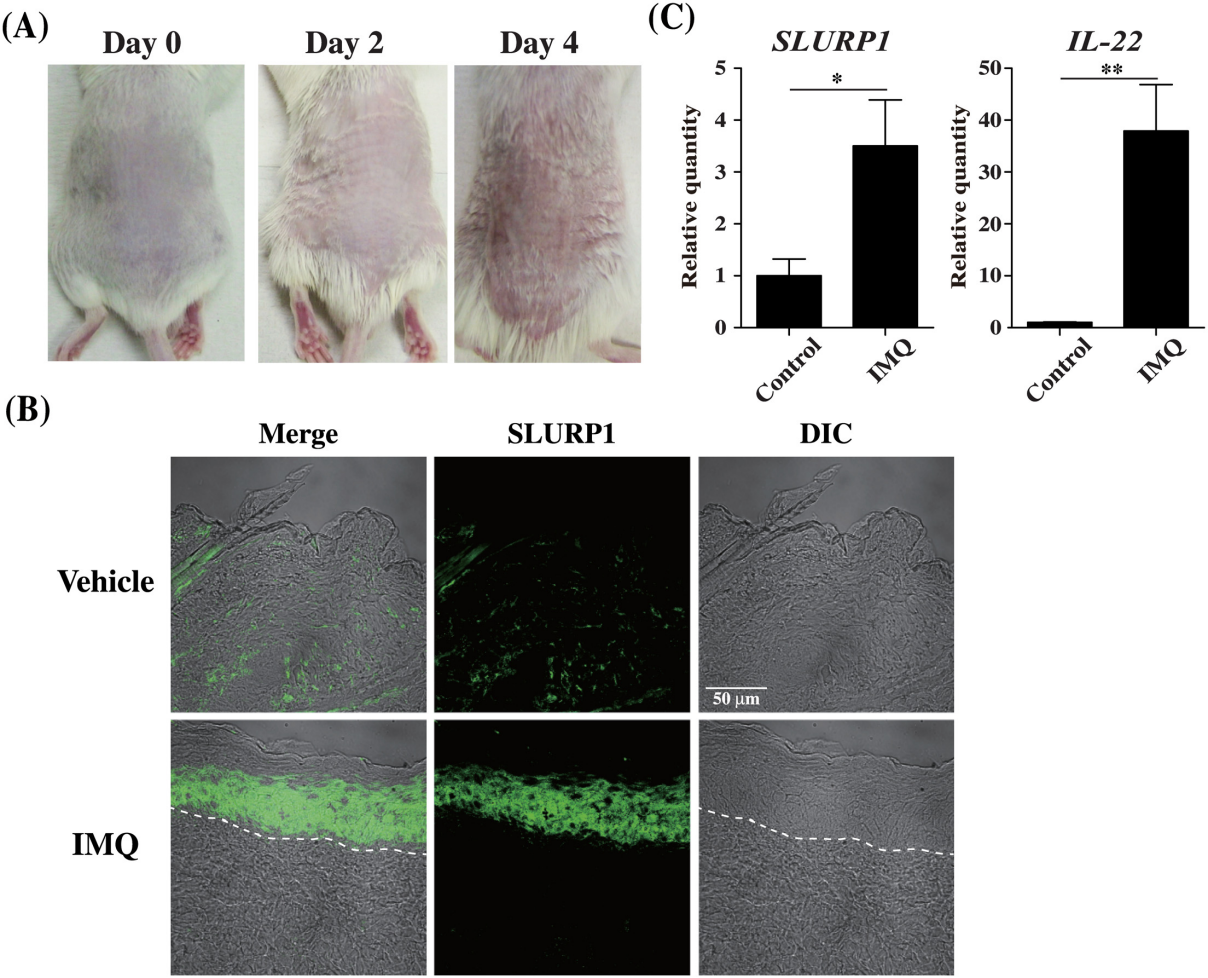
IMQ-induced psoriatic mouse skin has an abundance of SLURP1. IMQ or control cream was applied daily to the shaved backs for BALB/c mice. (A) Phenotypical presentation of mouse back skin after 2 or 4 days of IMQ treatment. (B) Immunofluorescent staining of vehicle or IMQ cream-treated mouse skin using an anti-SLURP1 antibody. Dashed lines indicate the border between the epidermis and dermis. Scale bars = 50 μm. The experiments were repeated six times with similar results. (C) Quantification of *IL-22* and *SLURP1* mRNA expression in skin from mice treated for 2 days with IMQ. Bars depict the mean ± S.D. (n = 6) of fold-changes in mRNA copy number normalized to GPADH and quantified relative to control. *p < 0.05, **p < 0.01 vs. control (Student’s t-test).

IMQ-treated mice develop a psoriasis-like skin disorder [25]. We assessed SLURP1 expression in skin from the backs of mice treated with IMQ for 2 or 4 consecutive days. As shown in Fig 1A, we found that IMQ-treated skin developed signs of erythema, scaling and thickening over the course of treatment. Acanthosis is caused by keratinocyte hyperproliferation and scaling is caused by altered epidermal differentiation; both are seen in psoriatic skin lesions and were observed in the IMQ-treated mice (data not shown). Within the psoriasis-like skin lesions, SLURP1 protein expression was significantly increased in hyperproliferative keratinocytes (Fig 1B), and quantitative real-time PCR revealed corresponding elevation of *SLURP1* mRNA expression (Fig 1C). In addition, the induction of IL-22, which appears to contribute to keratinocyte proliferation and epidermal hyperplasia, was also detected in the same skin samples (Fig 1C). The result in Fig 1B was highly reproducible, as indicated by the same results with other five mice (S1 Fig).

### SLURP1 expression is enhanced by IL-22 in NHEKs

**Fig 2:**
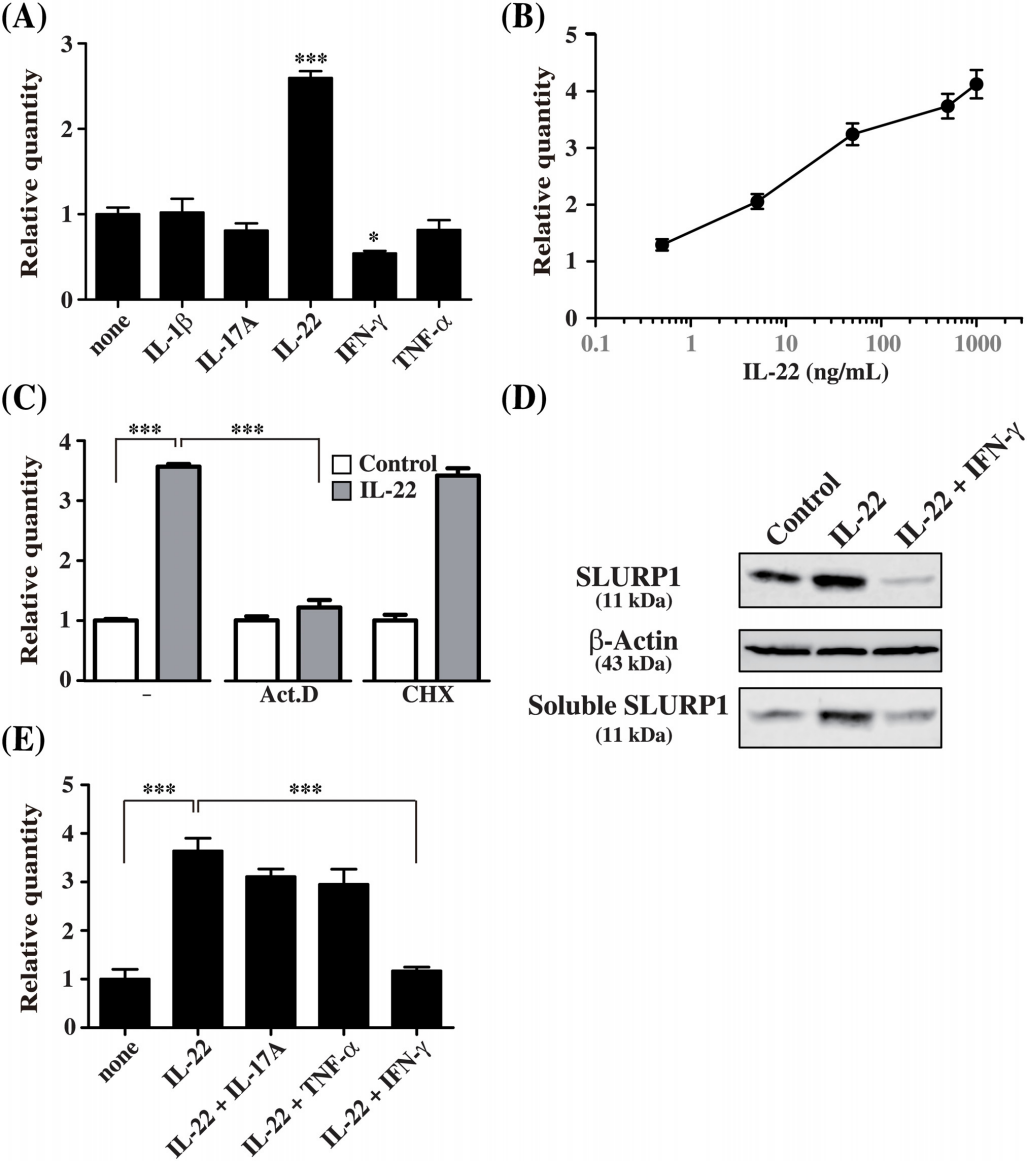
SLURP1 expression is directly and transcriptionally regulated through IL-22. (A, B) NHEKs were stimulated 24 h with the indicated panel of cytokines (50 ng/ml) (A) or with 0.5, 5, 50, 500 and 1000 ng/ml IL-22 (B), after which *SLURP1* mRNA levels were measured using quantitative real-time PCR. (C) NHEKs were stimulated with IL-22 or left untreated for 24 h in the presence of actinomycin D (Act. D), cycloheximide (CHX) or the control solvent (DMSO), after which *SLURP1* expression was analyzed using real-time PCR. (D, E) NHEKs were stimulated for 24 h with IL-22 or a combination of IL-22 plus IL-17A, TNF-α or IFN-γ. *SLURP1* expression was then analyzed using real-time PCR (D) and western blotting (E). Bars depict the mean ± S.D. (n = 6). *p < 0.05, ***p < 0.001 (one-way ANOVA).

Several inflammatory cytokines, including IL-1β, IL-17A, IL-22 and TNF-α, are reportedly up-regulated in psoriatic lesions [2–4]. To identify endogenous factors involved in the regulation of *SLURP1* gene expression, NHEKs were stimulated using the panel of cytokines shown in Fig 2A. Expression of *SLURP1* mRNA was enhanced by IL-22, but not IL-1β, IL-17A, IFN-γ or TNF-α. Moreover, the stimulatory effect of IL-22 was dose-dependent (Fig 2B) and was inhibited by simultaneous addition of actinomycin D, but not by cycloheximide (Fig 2C). Expression of SLURP1 was enhanced by 50 ng/mL IL-22, and the protein was detected as a secreted form in the culture supernatant (Fig 2D). Taken together, these results suggest that *SLURP1* is a direct target of IL-22 and that the increase in SLURP1 protein expression was transcriptionally regulated.

IL-17A and TNF-α have been shown to act cooperatively with IL-22 to enhance AMP expression (i.e., β Defensin2, S100A7/psoriacin and other S100 family peptides), chemokine expression (e.g., CXCL10, CCL5) and proinflammatory Th22 responses in T cells [23,27]. On the other hand, IFN-γ reportedly suppresses expression of SLURP1 [28]. To examine these effects qPCR analysis was performed for SLURP1, CCL5 and S100A7. As shown in Fig 2E, IL-22-induced *SLURP1* mRNA expression was not cooperatively enhanced by IL-17A or TNF-α, but it was suppressed with IFN-γ. This suppression was also confirmed at the protein level, as IL-22 did not increased SLURP1 levels in the supernatants of cultures treated with IFN-γ (Fig 2D).

**Fig 3:**
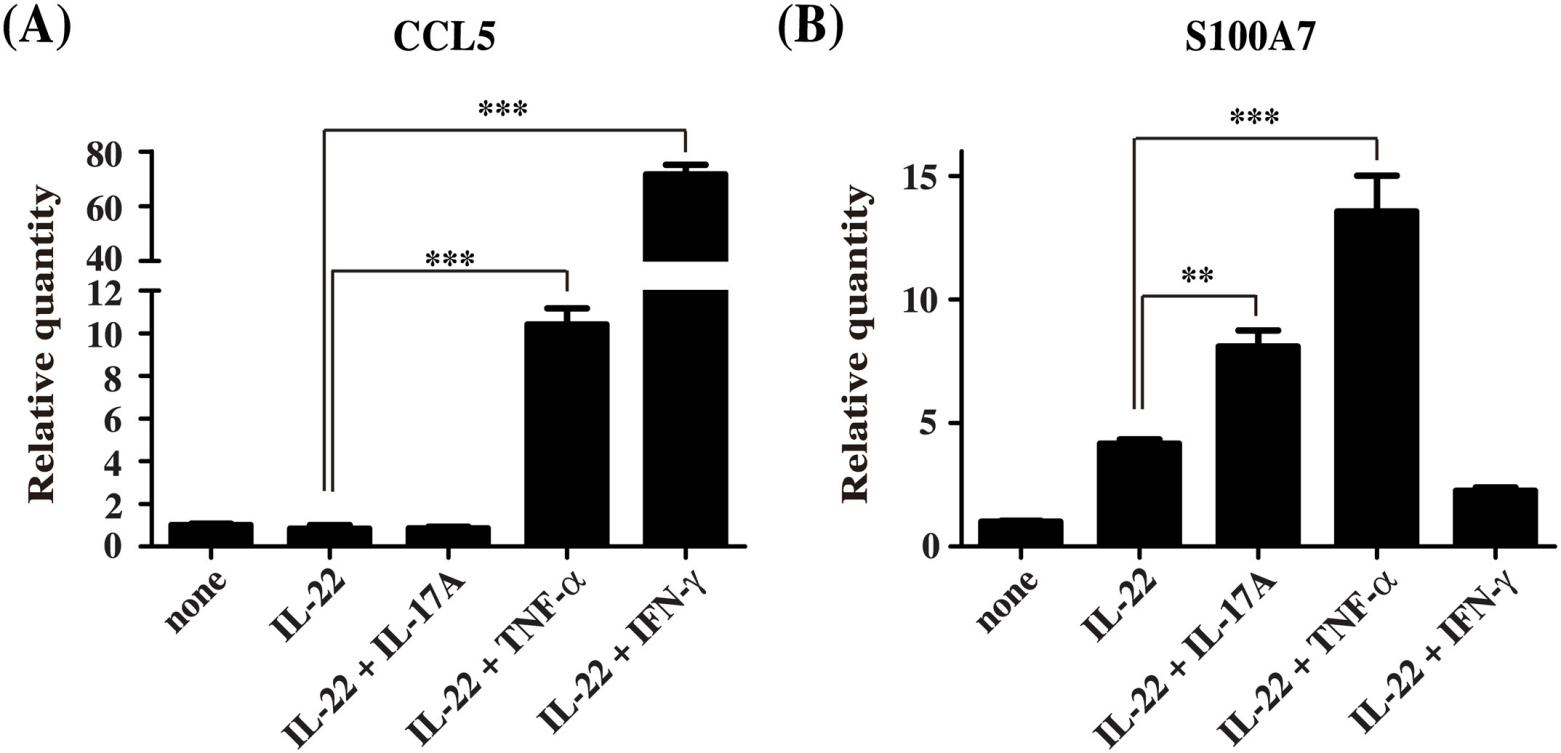
Cooperative role of TNF-α and IL-17 with IL-22 on S100A7 expression. (A, B) NHEKs were stimulated for 24 h with IL-22 or a combination of IL-22 plus IL-17A, TNF-α or IFN-γ. RNA was isolated from the cells and subjected to quantitative real-time PCR analysis for *CCL5* (A) *and S100A7* (B) expression were then analyzed using real-time PCR. Bars depict the mean ± S.D. (n = 6). **p < 0.01, ***p < 0.001 (one-way ANOVA).

TNF-α and IFN-γ stimulation significantly increased CCL5 expression (Fig 3A) and IL-22 in combination with IL-17A or TNF-α augmented the effect of IL-22 on S100A7 expression (Fig 3B). These results clearly demonstrated both IL-17A and TNF-α are active. However, similar to SLURP1 expression, IFN-γ combined with IL-22 abolished the effect of IL-22 on S100A7 expression (Fig 3B). These results indicate that IFN-γ somehow affects IL-22-induced gene expression.

### SLURP1 expression is under control of the IL-22-STAT3 axis

**Fig 4:**
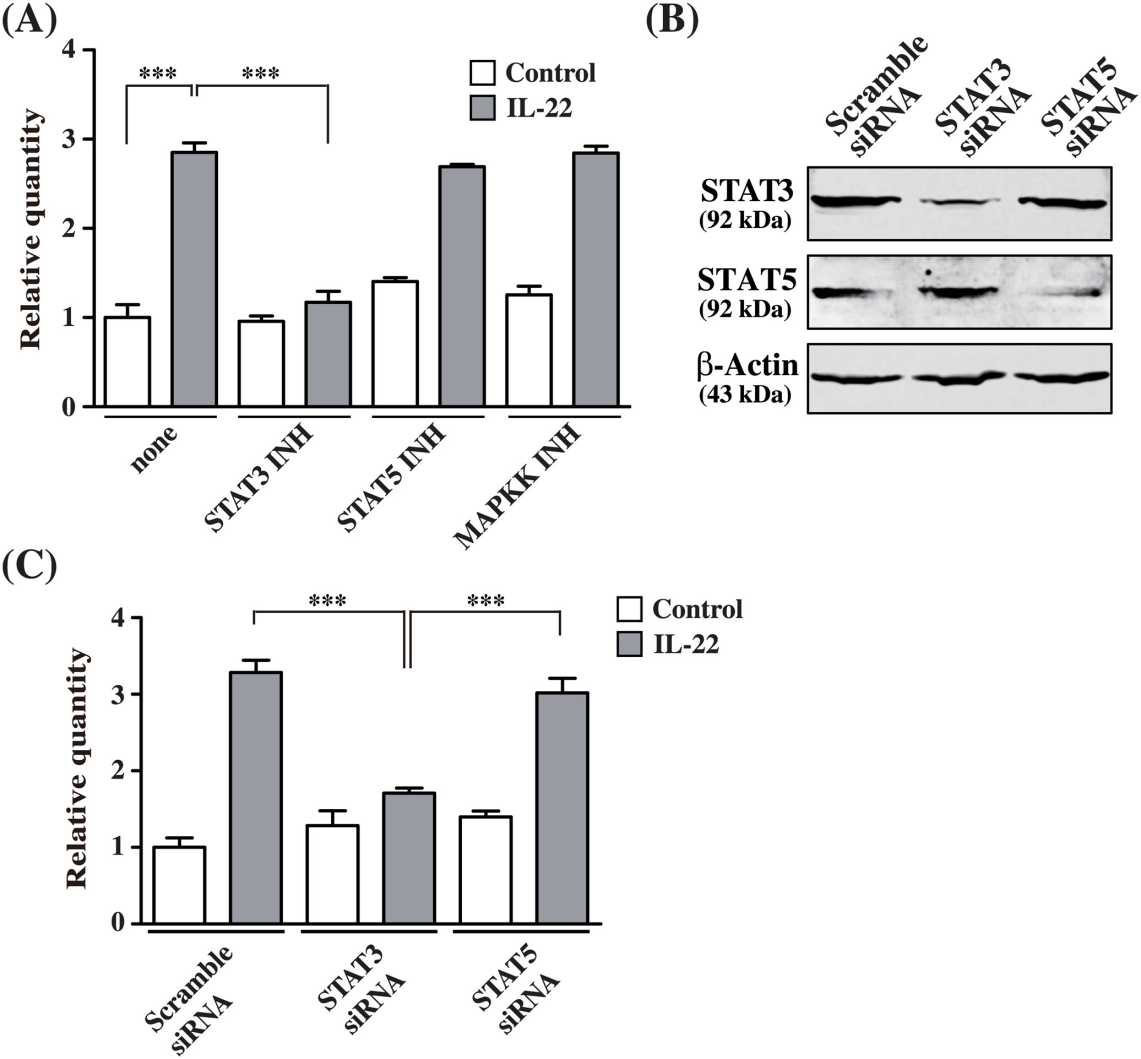
STAT3 knockdown abolishes IL-22-induced SLURP1 induction in NHEKs. (A) NHEKs were incubated for 24 h with IL-22 (50 ng/ml) in the presence or absence of the MAP kinase kinase inhibitor PD98059 (30 μM), the STAT3 inhibitor S3I-201 (50 μM) or the STAT5 inhibitor 573108 (100) μM), after which *SLURP1* expression was analyzed using real-time PCR. As a control, 0.1% (v/v) DMSO was used. Bars depict the mean ± S.D. (n = 6). (B, C) NHEKs were transfected with 100 nM scrambled control, STAT3 or STAT5 siRNA. After incubating the transfectants for 48 h, IL-22 was added to a final concentration of 50 ng/ml, and the cells were harvested 24 h later. (B) Cell lysates (30 μg of total protein/lane) were subjected to SDS-PAGE, and immunoblots were probed with anti-STAT3, anti-STAT5 or anti-β-actin antibody. The experiments were repeated six times with similar results. (C) Quantitative real-time PCR analysis of *SLURP1* mRNA. Bars depict the mean ± S.D. (n = 6). ***p < 0.001 (one-way ANOVA).

To investigate the signal transduction pathway leading from IL-22 stimulation to enhanced *SLURP1* transcription, IL-22-stimulated NHEKs were treated with a panel of signal transduction inhibitors. The enhancement of *SLURP1* mRNA expression was completely blocked by the STAT3 inhibitor S3I-201, but was unaffected by the STAT5 inhibitor 573108 or the MAP kinase kinase inhibitor PD980589 (Fig 4A). To further confirm this result, NHEKs were transfected with siRNA targeting STAT3 or STAT5. Expression of the respective proteins was greatly suppressed in cells expressing STAT3 or STAT5 siRNA (Fig 4B). Moreover, IL-22-induced expression of *SLURP1* mRNA was also suppressed in the STAT3 knockdown cells (Fig 4C). On the other hand, *SLURP1* induction was unaffected by STAT5 knockdown. These results suggest that *SLURP1* expression is under the control of the IL-22-STAT3 axis.

### Antibacterial activities of SLURP1 against *S*. *aureus*

**Fig 5:**
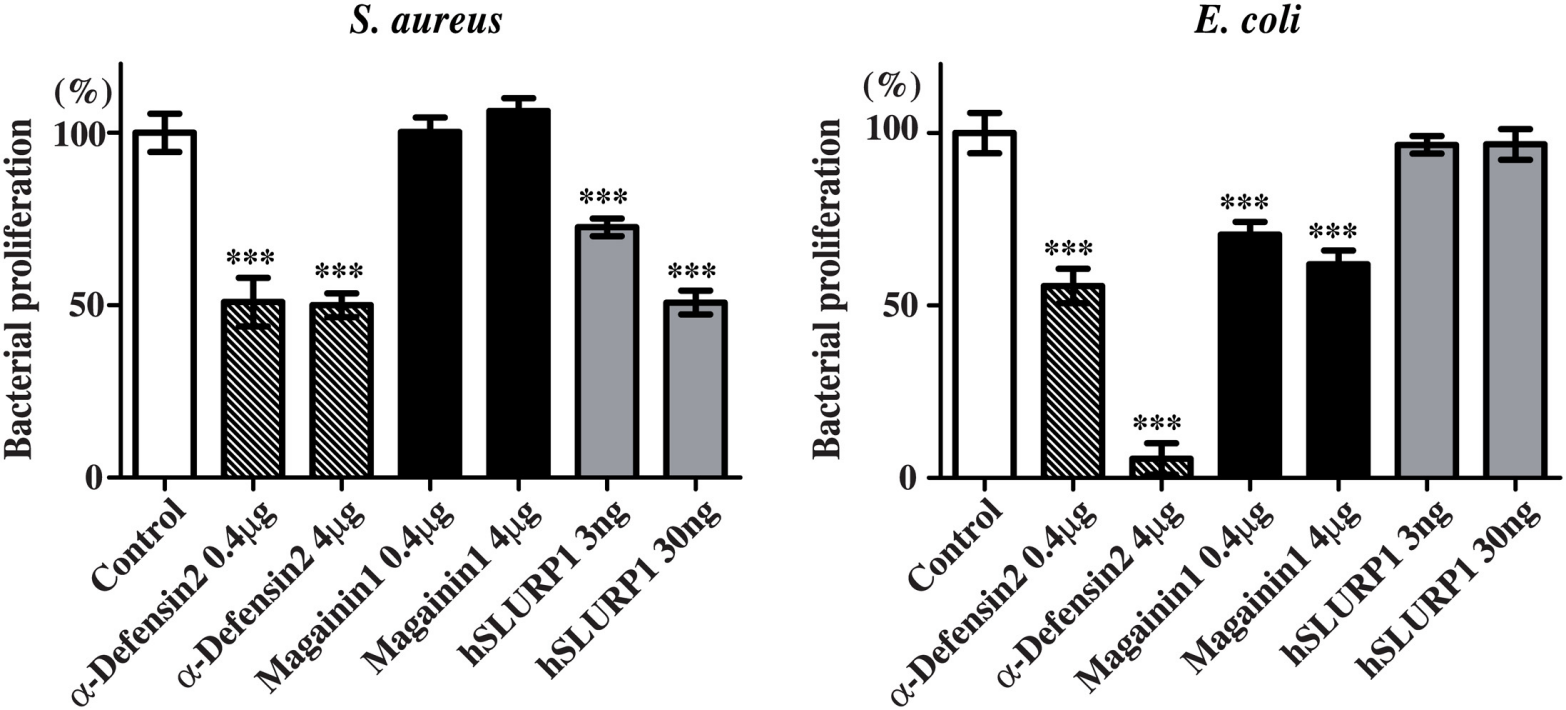
SLURP1 exhibits an antibacterial activity against *S*. *aureus*. Mid-exponential growth phase *S*. *aureus* amd *E*. *coli* were incubated for 18 h with α-defensin2, magainin1 or rhSLURP1 purified from culture supernatants. After the incubation, bacterial proliferation was assessed based on the change in the OD_620_. The results are presented as percentages of growth relative to the untreated control. This assay was repeated in six independent experiments performed in triplicates. ***p < 0.001 (one-way ANOVA).

IL-22 induces production of various AMPs, including β-defensin2, S100A7 and LL37, in regions of skin inflammation [23,24]. Because it has been reported that prostate and testis expressed protein (PATE), which is a secreted protein containing a Ly6 domain, has antibacterial activity [29], we investigated the antibacterial activity of partially purified rhSLURP1 secreted from SLURP1-transfected RK13 cells. As shown in Fig 5, SLURP1 dose dependently suppressed proliferation of the gram-positive bacterium *S*. *aureus* but not gram-negative bacterium *E*. *coli*. Although antibacterial activity of α-defensin2 to gram-negative bacterium *E*. *coli* was quite high, the suppressive effect of α-defensin2 on *S*. *aureus* proliferation was almost similar to that of SLURP1 (Fig 5). Magainin1 which does not kill gram-positive bacteria had no effect on proliferation of *S*. *aureus* [30], however magainin1 significantly suppressed proliferation of gram-negative bacterium *E*. *coli* at the same dose used for gram-positive bacteria (Fig 5). These results clearly demonstrate that SLURP1 is effective in suppression of proliferation in the gram-positive bacterium *S*. *aureus* but not gram-negative bacterium *E*. *coli*. More concentration of SLURP1 protein may completely suppress the proliferation of *S*. *aureus*, however the physiological concentration of SLURP1 has been reported around 10 ng/ml [28].

## Discussion

In this study, we demonstrated that 1) SLURP1 expression is enhanced in the skin of IMQ-induced psoriatic model mice; 2) expression of *SLURP1* mRNA is induced by IL-22 in keratinocytes; and 3) this up-regulation of *SLURP1* transcription is completely suppressed by a STAT3 inhibitor or by siRNA targeting STAT3, which is a major transcriptional factor downstream of IL-22. Wolk and colleagues [19] previously showed that IL-22 is present at high levels in the skin of patients with psoriasis, but is undetectable in healthy skin. Correspondingly, psoriasis patients exhibit elevated plasma IL-22 levels that correlate with disease severity [3,19]. It has also been reported that STAT3 is strongly expressed in keratinocytes within psoriatic skin lesions and that levels are especially high in the nucleus, where it participates in the hyper-proliferation of keratinocytes and inflammatory infiltration [31]. Consistent with these observations, our results suggest that SLURP1 expression is increased within psoriatic lesions. The increased expression of SLURP1 in hyperproliferative keratinocytes within psoriatic lesions is consistent with the increased turnover of the epidermis in psoriasis because SLURP1 accelerates differentiation and apoptosis in keratinocytes [10]. Furthermore, SLURP-1 expressed in keratinocytes was recently reported to regulate wound healing [32]. These findings suggest the possibility that the increased SLURP1 expression is one of compensatory reactions to maintain epidermal homeostasis in psoriatic lesions.

We also found that even a low concentration of rhSLURP1 significantly suppressed the growth of *S*. *aureus* (Fig 5). Importantly, *S*. *aureus* is detected in about 50% of skin lesions in patients with psoriasis [14,15], and is associated with a higher PASI score [16]. These observations indicate that SLURP1 is not only a mediator that regulates keratinocyte differentiation and apoptosis, it may also be an important factor that reduces the disease activity of psoriasis by suppressing the growth of *S*. *aureus*.

When applied in combination with IL-22, neither IL-17A nor TNF-α acted cooperatively with IL-22 to enhance SLURP1 expression, while IFN-γ completely abolished IL-22-induced SLURP1 expression (Fig 2D). It has been reported that IL-17A and IL-17F do not alter expression of the IL-22 receptor [23]. On the other hand, TNF-α is known to enhance the effect of IL-22 on keratinocytes by increasing expression of both the IL-22 receptor and STAT3 [22]. In the present study, IL-22 increased *SLURP1* expression in a dose-dependent manner via a STAT3-mediated pathway. In addition, we also found that TNF-α increased expression of IL-22RA1 (data not shown). However, the fact that the TNF-α-mediated enhancement of IL-22 receptor expression did not increase *SLURP1* expression indicates SLURP1 levels are tightly regulated by means other than STAT3 activation.

*SLURP1*-deficient mice exhibit severe PPK characterized by increased keratinocyte proliferation and water barrier defects that resemble MDM [11]. SLURP1 binds directly to α7-nAChRs, altering their function [10,12]. Although α7-nAChRs reportedly control the terminal differentiation of epidermal keratinocytes and skin homeostasis required for formation of the water barrier [13], α7-nAChR-deficient mice do not show symptoms of PPK. These observations suggest SLURP1 may regulate keratinocyte proliferation and differentiation through as yet unknown targets in addition to α7-nAChR.

We recently reported that SLURP1 increases ACh synthesis in T cells and attenuates T cell proliferation, and that these effects are abolished by methyllycaconitine, an α7-nAChR antagonist [33]. Those results indicate SLURP1 modulates the functional development of T cells via α7-nAChR-mediated pathways, which is consistent with the findings of Nizri et al. [34], who observed that nicotine suppresses antigen-induced T cell proliferation in wild-type mice but not in α7-nAChR deficient mice. They also reported that nicotine suppresses expression of Th1 and Th17 cytokines, such as TNF-α, IL-17 and IL22, and attenuates Th1 differentiation and expression of T-bet, a key transcriptional factor supporting Th1 development. Given that IL-22 is mainly produced by Th17, Th22 and Tc22 cells, SLURP1 may ameliorate psoriatic disease activity by modulating T cell function. Further investigation will be needed to determine whether SLURP1 itself suppresses Th1 or Th17 cytokine production by modulating T cell differentiation.

In sum, we found that SLURP1 expression is greatly increased within the skin lesions in a psoriatic mouse model, and that SLURP1 expression is increased via IL-22-STAT3 signaling in NHEKs. In addition, recombinant SLURP1 suppressed the growth of *S*. *aureus*, which is associated with a higher PASI score. These results suggest that interaction between SLURP1 and IL-22 may contribute to skin homeostasis as well as the pathogenesis of psoriasis.

## Supporting Information

S1 Fig Immunohistochemical analysis of SLURP1 in the skin of vehicle or IMQ cream treated mice. Immunofluorescent staining of vehicle or IMQ cream-treated mouse skin using an anti-SLURP1 antibody. Dashed lines indicate the border between the epidermis and dermis. Scale bars = 50 μm.
(TIF)
