# Supplementary figures and images for: IL-22/STAT3-Induced Increases in SLURP1 Expression within Psoriatic Lesions Exerts Antimicrobial Effects against Staphylococcus aureus
Source: PLoS One. 2015 Oct 16;10(10):e0140750. doi: 10.1371/journal.pone.0140750 (PMC4608685; doi:10.1371/journal.pone.0140750)

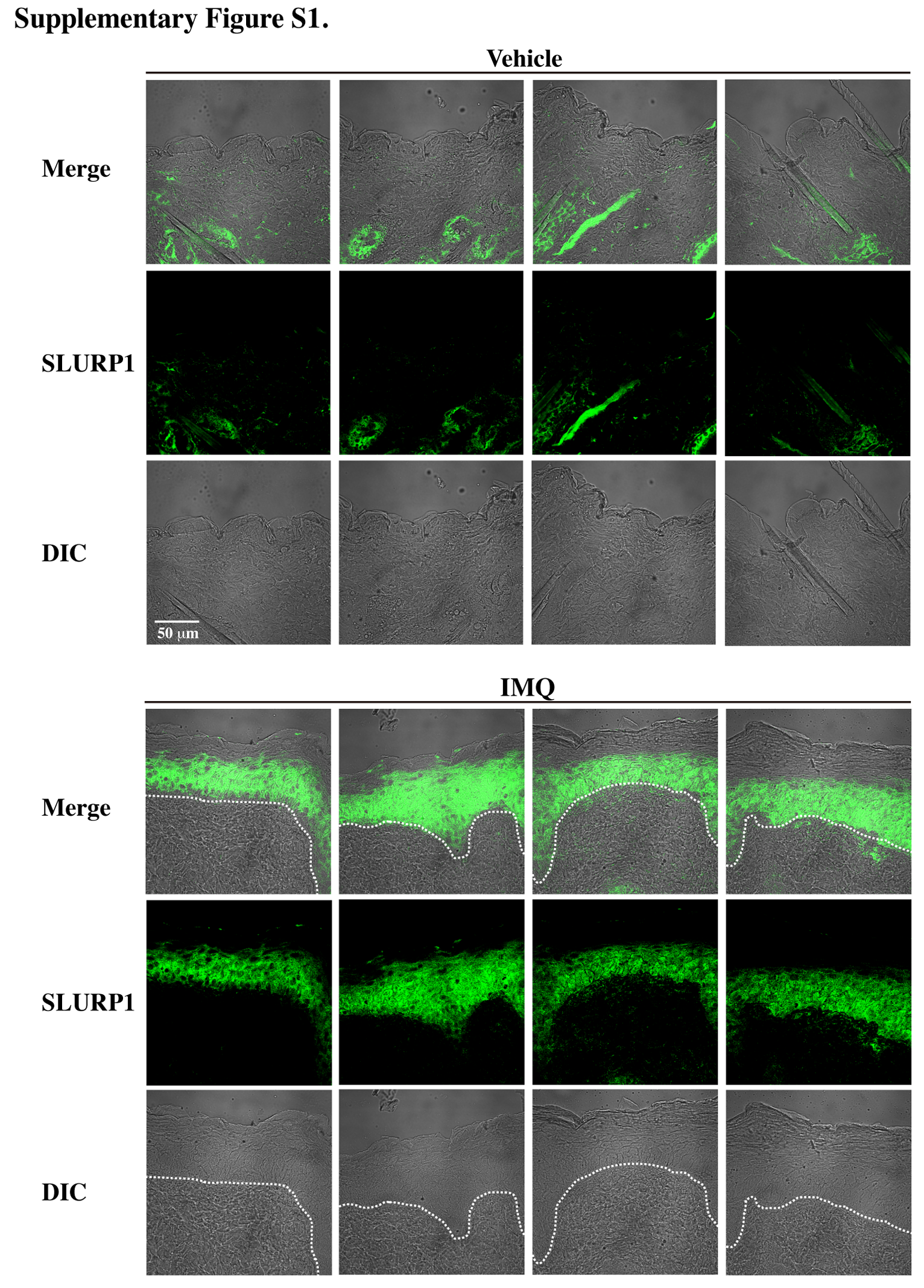

Supplement: S1 Fig — Immunofluorescent staining of vehicle or IMQ cream-treated mouse skin using an anti-SLURP1 antibody. Dashed lines indicate the border between the epidermis and dermis. Scale bars = 50 μm. (TIF) [file pone.0140750.s001.tif]
